# Adverse Childhood Experiences and Methylation of the *FKBP5* Gene in Patients with Psychotic Disorders

**DOI:** 10.3390/jcm9123792

**Published:** 2020-11-24

**Authors:** Błażej Misiak, Paweł Karpiński, Elżbieta Szmida, Tomasz Grąźlewski, Marcin Jabłoński, Katarzyna Cyranka, Joanna Rymaszewska, Patryk Piotrowski, Kamila Kotowicz, Dorota Frydecka

**Affiliations:** 1 Department of Psychiatry, Wroclaw Medical University, Pasteura 10 Street, 50-367 Wroclaw, Poland; joanna.rymaszewska@umed.wroc.pl (J.R.); patryk.piotrowski@umed.wroc.pl (P.P.); kamila.kotowicz@gmail.com (K.K.); dfrydecka@gmail.com (D.F.); 2 Department of Genetics, Wroclaw Medical University, Marcinkowskiego 1 Street, 50-368 Wroclaw, Poland; polemiraza@poczta.fm (P.K.); e.szmida@gmail.com (E.S.); 3 Laboratory of Genomics & Bioinformatics, Institute of Immunology and Experimental Therapy, Polish Academy of Sciences, Weigla 12 Street, 53-114 Wroclaw, Poland; 4 Department of Psychiatry, Pomeranian Medical University, Broniewskiego 26 Street, 71-460 Szczecin, Poland; tgrazlewski@gmail.com (T.G.); marcinjablonski2@wp.pl (M.J.); 5 Department of Psychiatry, Jagiellonian University, Kopernika 21a Street, 31-501 Cracow, Poland; katarzyna.cyranka@gmail.com

**Keywords:** epigenetics, early-life stress, childhood trauma, childhood maltreatment, cortisol

## Abstract

Altered methylation of the *FKBP5* gene has been observed in various mental disorders and attributed to the effects of adverse childhood experiences (ACEs). However, the level of *FKBP5* methylation has not been investigated in patients with psychotic disorders. Therefore, in this study we aimed to determine the *FKBP5* methylation in patients with psychosis and controls, taking into account the effects of ACEs. Participants were 85 patients with psychotic disorders, including first-episode psychosis (FEP) patients and acutely relapsed schizophrenia (SCZ-AR) patients, as well as 56 controls. The level of four CpG sites at the *FKBP5* gene was determined in the peripheral blood leukocytes using pyrosequencing. After controlling for potential confounding factors, the level of *FKBP5* methylation at one out of four tested CpG sites was significantly lower in FEP patients compared to other groups of participants. Significant main effects of parental antipathy and sexual abuse on the level of *FKBP5* methylation were observed at the differentially methylated CpG site. Participants reporting this category of ACEs had significantly lower levels of *FKBP5* methylation at this CpG site. Lower levels of *FKBP5* methylation were associated with better cognitive performance and higher functional capacity in patients with psychosis. In controls, lower methylation of *FKBP5* was related to worse performance of immediate memory and language skills. Our findings suggest that hypomethylation of the *FKBP5* appears at early stages of psychosis and might be associated with a history of ACEs as well as less severe clinical manifestation.

## 1. Introduction

Adverse childhood experiences (ACEs), including physical and sexual abuse, emotional abuse and neglect as well as parental loss, represent well-documented risk factors for psychosis [1,2,3]. Moreover, a history of ACEs has been associated with more severe symptomatic manifestation [4,5], cognitive impairment [6] and worse treatment outcomes [7,8]. To date, various biological mechanisms have been proposed to explain the association between ACEs and susceptibility to psychosis. One of them is related to dysregulation of the hypothalamic-pituitary-adrenal (HPA) axis. Indeed, individuals with a history of ACEs have a heightened negative reaction to distressing experiences later in life [9], elevated cortisol levels over time [10] and blunted HPA axis responses on stress reactivity tests [11]. Moreover, it has been demonstrated that glucocorticoid secretion increases dopaminergic activity in various brain regions, especially the mesolimbic system [12,13]. To date, several HPA axis dysregulations have been observed in patients with schizophrenia and first-episode psychosis (FEP), including pituitary enlargement [14], elevated morning cortisol levels [15], blunted cortisol awakening response together with flattened HPA axis response to stress [16,17,18].

A recent meta-analysis demonstrated poor concordance between naturally-occurring stressors and the HPA axis dysfunction in patients with psychosis [19]. Apart from certain methodological considerations, it should be noted that exposure to ACEs is not a risk factor specifically associated with the psychosis spectrum. Moreover, early-life stress does not always lead to the development of unfavourable mental health outcomes. One of the potential moderators of this association might be related to the impact of genetic and epigenetic factors [20,21]. Indeed, epigenetic processes have been demonstrated to act in transducing environmental experiences to both the genome and brain structure modifications, potentially underlining the association between childhood trauma and the development of psychosis as well as its psychopathological features and biological correlates [22].

The gene by which ACEs interactions that might impact a risk of various neuropsychiatric disorders, including psychosis, has been described as the *FKBP5* gene (for review see [20,23]). This gene encodes the FK506-binding protein 51 (FKBP51) that is a heat shock protein acting as a co-chaperone for the glucocorticoid receptors [24]. Expression of the *FKBP5* gene is strongly stress-responsive and higher levels of FKBP51 cause diminished negative feedback regulation of the HPA axis, thus prolonging stress response through longer reduction of cortisol secretion [25]. The *FKBP5* polymorphisms have been shown to be associated with psychosis after the inclusion of ACEs as the confounding factor [26]. Moreover, ACEs have been demonstrated to interact with variation in the *FKBP5* gene affecting clinical manifestation of psychosis and cognitive performance [27,28]. It is also important that exposure to ACEs has been associated with lower methylation of the *FKBP5* gene [29,30].

Although higher expression of the *FKBP5* gene has been observed in patients with schizophrenia [31], methylation of this gene has not been investigated in this group of patients. Moreover, the association between exposure to ACEs and the level of *FKBP5* methylation in patients with psychotic disorders remains unknown. Therefore, in this study, we aimed to assess the level of *FKBP5* methylation in patients with FEP, acutely relapsed schizophrenia (SCZ-AR) patients, and healthy controls. Furthermore, we investigated the association between clinical manifestation, cognitive performance and a history of ACEs in this group of patients.

## 2. Materials and Methods

### 2.1. Participants

Participants were represented by 85 inpatients with schizophrenia-spectrum disorders and 56 healthy controls. They overlapped with samples reported in detail by our previous publications [32,33,34]. There were 40 patients with FEP and 45 acutely-relapsed patients with SCZ-AR. Patients were recruited at two clinical sites (Department of Psychiatry, Wroclaw Medical University, Wroclaw, Poland and Department of Psychiatry, Pomeranian Medical University, Szczecin, Poland). The Diagnostic and Statistical Manual of Mental Disorders, fourth edition (DSM-IV) criteria, validated by the Operational Criteria for Psychotic Illness (OPCRIT) checklist, were used to establish a clinical diagnosis [35]. Patients with FEP met the DSM-IV criteria for the following diagnoses: schizophrenia (*n* = 14), delusional disorder (*n* = 1), schizoaffective disorder (*n* = 5), schizophreniform disorder (*n* = 7) and brief psychotic disorder (*n* = 13). In turn, all SCZ-AR patients met the DSM-IV criteria of schizophrenia. Most patients were receiving antipsychotic treatment on the day of recruitment (there were two antipsychotic-naïve FEP patients). The total chlorpromazine equivalent dosage (CPZeq) was 380.6 ± 211.6 mg/day. Healthy controls were recruited through advertisements and had a negative family history of psychotic and mood disorders in first- and second-degree relatives. Both groups of participants were matched for age, sex and the level of parental education. The latter one represented a proxy measure of socioeconomic status. The study protocol was approved by the Ethics Committee at Wroclaw Medical University (Poland) and all participants gave written informed consent.

### 2.2. Clinical Assessment

The following measures were used to record symptomatic manifestation on the day of recruitment: (1) the Positive and Negative Syndrome Scale (PANSS) [36]; (2) the Montgomery–Asberg Depression Rating Scale (MADRS) [37]; (3) the Young Mania Rating Scale (YMRS) [38] and (4) the Global Assessment of Functioning (GAF) [39]. Cognitive performance was assessed by the Repeatable Battery for the Assessment of Neuropsychological Status (RBANS) [40]. It consists of 12 tasks scoring the following domains of cognitive performance: (1) immediate memory (list learning and story memory); (2) visuospatial/constructional functions (figure copy and line orientation); (3) language (picture naming and semantic fluency); (4) attention (digit span and coding); and (5) delayed memory (list recall, list recognition, story memory and figure recall).

The Childhood Experience of Care and Abuse Questionnaire (CECA.Q) was administered to assess a history of childhood maltreatment [41]. The CECA.Q is a retrospective self-report that records various childhood adversities appearing before the age of 17 years. These include parental loss, parental antipathy and neglect, physical abuse as well as sexual abuse. It has been validated in the population of patients with psychosis and has good psychometric properties [42].

### 2.3. Sampling of Biological Material

Two venous blood samples were collected after overnight fasting between 7 a.m. and 9 a.m. One of them was centrifuged to obtain serum that was stored in aliquots at −80 °C. Serum levels of cortisol were determined using electrochemiluminescence analysis (Cobas e411 analyser, Roche). DNA was obtained from peripheral blood leukocytes using the Prepito DNA Blood250 Kit according to the manufacturer’s protocol.

### 2.4. Assessment of DNA Methylation

**Figure 1:**
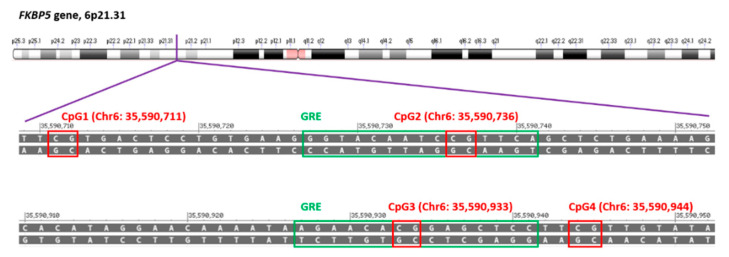
Location of CpG sites tested in the present study according to the Genome Reference Consortium Human Build 38 patch release 7 primary assembly in the National Center of Biotechnology Information Variation Viewer. Selected CpG sites were marked with red boxes. Location and sequence of glucocorticoid response elements (GRE) was marked with green boxes.

Four CpG sites were selected for pyrosequencing based on their proximity to glucocorticoid response elements (GREs) (Figure 1). Bisulfite treatment was carried out using 1400 ng of a sample genomic DNA and the EZ DNA Methylation-Direct kit (Zymo Research, Orange, CA, USA). This process deaminates unmethylated cytosine residues to uracil leaving methylated cytosine residues unchanged. To perform polymerase chain reactions (PCR), 42 ng of bisulfite-modified DNA was used as template. The PCR reactions were performed in a total volume of 50 µL for 35 cycles using Roche Diagnostic Corporation (Indianapolis, IN, USA), FastStart High-Fidelity Taq DNA Polymerase (1.0U), MgCl2 solution (3.5 mM), deoxynucleotides (0.2 mM), sense primer (0.24 uM), antisense primer (0.18 µM), with denaturation at 95 °C for 30 s, annealing for 45 s at 57 °C and 53 °C, and extension at 72 °C for 1 min.

The following sets of primers were used: (1) sense primer: 5′-GGATAATAATTTGGAGTTATAGTGTAGGT-3′, anti-sense primer: 5′-CAAAACTTATTCCCTTATTTATTCCTAAAC-3′ and sequencing primer: 5′-ATTTGGAGTTATAGTGTAGGTTT-3′ (PCR product: 192 bp, annealing temperature: 57 °C) and (2) 5′-sense primer: AAAAGTTGATATATAGGAATAAAATAAGA-3′, anti-sense primer: 5′-TATTTATTCATTATCAAATTTATCTCTTAC-3′ and sequencing primer: 5′-ATATAGGAATAAAATAAGAAT-3′ (PCR product: 130 bp, annealing temperature: 53 °C). All PCR products were electrophoresed on 1% agarose gel, stained with ethidium bromide, and visualized for appropriate and pure product before proceeding with all analyses using the Bio-Rad Laboratories (Hercules, CA, USA) Gel-Doc UV illuminator. Methylation percentage of each CpG was determined using the Qiagen (Valencia, CA, USA) Pyromark Q96 ID pyrosequencer and sequencing primers, according to the manufacturer’s recommendations.

### 2.5. Statistics

Bivariate comparisons were performed using the Mann–Whitney U test or Student’s *t*-test (depending on data distribution) and the chi-squared test. One-way analysis of variance (ANOVA) was used to test differences in continuous variables between FEP patients, SCZ-AR patients and healthy controls. In the case of significant results of one-way ANOVA, post-hoc tests were used (Bonferroni test or Games–Howell test, depending on homogeneity of variance). Correlations were tested using the Spearman’s rank correlation coefficients. Differences in the level of *FKBP5* methylation were further assessed using the analysis of co-variance (ANCOVA). The following covariates were considered and further selected based on the analysis of correlations with the *FKBP5* methylation levels: age, sex, body mass index (BMI), cigarette smoking status, cortisol levels and CPZeq. All of these variables, except for CPZeq, have been associated with the levels of *FKBP5* methylation in previous studies [43,44]. In turn, CPZeq was a proxy measure of exposure to antipsychotics which have been found to impact DNA methylation [45]. Independent variables were represented by the participants status (FEP, SCZ-AR or healthy controls) and a history of specific childhood adversities. Results of statistical analysis were considered statistically significant if the *p*-value was <0.05. The Statistical Package for Social Sciences, version 20 (SPSS Inc., Chicago, IL, USA) was used to perform statistical analyses.

## 3. Results

General characteristics of participants were presented in Table 1. There were significant between-group differences in terms of age, education, BMI, cigarette smoking rates, cognitive performance on all RBANS domains and cortisol levels. Additionally, patients with SCZ-AR had significantly longer illness duration, higher scores of negative symptoms, lower GAF scores and greater CPZeq.

**Figure 2:**
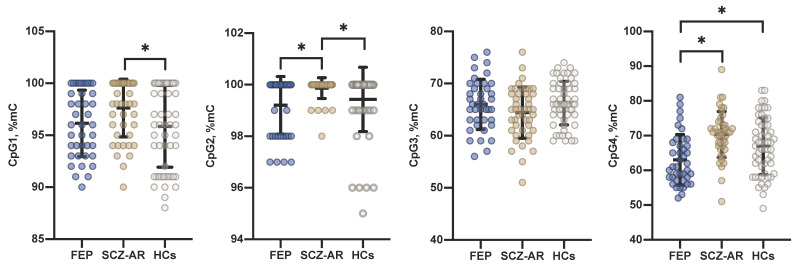
The levels of *FKBP5* methylation in patients with first-episode psychosis (FEP), acutely relapsed schizophrenia patients (SCZ-R) and healthy controls (HCs). Horizontal lines and error bars represent mean and standard deviation. * *p* < 0.05.

The levels of cortisol were significantly higher in patients with psychotic disorders compared to controls. There were no significant between-group differences between the whole group of patients and healthy controls in the levels of *FKBP5* methylation (Table 1). However, further stratification of the sample revealed the following significant differences (post-hoc tests): (1) higher CpG1 methylation in SCZ-AR patients compared to healthy controls (*p* = 0.026); (2) higher CpG2 methylation in SCZ-AR patients compared to FEP patients (*p* = 0.002) and healthy controls (*p* = 0.042) and (3) lower CpG4 methylation in FEP patients compared to SCZ-AR patients (*p* < 0.001) and healthy controls (*p* = 0.033) (Figure 2).

**Figure 3:**
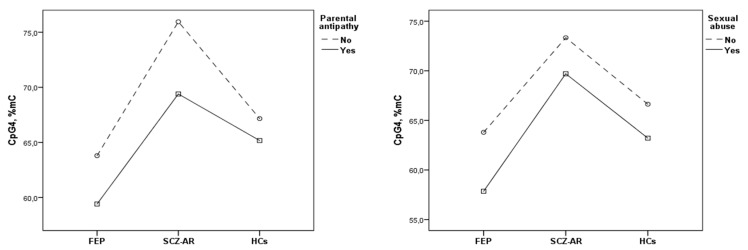
The association between a history of parental antipathy and sexual abuse with methylation levels at CpG4 in patients with first-episode psychosis (FEP), acutely relapsed schizophrenia patients (SCZ-R) and healthy controls (HCs). Estimated marginal means were shown.

Bivariate correlations between the FKBP5 methylation levels and potential confounding factors were presented in Table A1. Based on this analysis, the following factors were associated with the FKBP5 methylation: (1) BMI for CpG1; (2) age, sex and cortisol levels for CpG2; (3) sex, cigarette smoking status, BMI and cortisol levels for CpG3 and (4) sex, BMI and CPZeq for CpG4. These variables were included as covariates in the ANCOVA (Table 2). There were significant main effects of diagnostic group (FEP vs. SCZ-AR vs. healthy controls) on the level of CpG4 methylation in the models testing all categories of adverse childhood experiences. These effects were not significant in the models that included methylation of CpG1, CpG2 and CpG3 as dependent variables. There were also significant main effects of parental antipathy and sexual abuse on the level of CpG4 methylation. More specifically, parental antipathy and sexual abuse were related to lower CpG4 methylation in all participants (Figure 3). Regarding covariates, significant main effects of sex were found in all models that included the levels of CpG3 and CpG4 methylation. In turn, BMI was associated with the level of CpG4 methylation in the majority of ANCOVA models, except for the one that included a history of parental neglect.

Correlations between clinical variables and the levels of *FKBP5* methylation are shown in Table 3. Higher CpG2 methylation was associated with significantly lower scores of immediate memory, visuospatial/constructional abilities, attention and delayed memory as well as the GAF in patients with psychosis. However, the correlation with visuospatial/constructional abilities was significant only in patients with FEP. Similarly, higher CpG4 methylation was associated with lower scores of the RBANS (except for the scores of language and visuospatial/constructional abilities) and the GAF in the group of patients with psychosis. In healthy controls, higher methylation of CpG4 was related to higher scores of the RBANS language and immediate memory domains.

## 4. Discussion

To our knowledge, this is the first study investigating the level of *FKBP5* methylation in patients with psychotic disorders with respect to a history of ACEs. Our findings indicate that only patients at early stages of illness may show decreased levels of *FKBP5* methylation at one of four tested CpG islands (CpG4). This study also demonstrated the main effects of sexual abuse and parental antipathy on the level of CpG4 methylation that appeared to be lower in participants reporting these categories of ACEs. This observation is in agreement with several previous studies reporting lower *FKBP5* methylation in various clinical and non-clinical populations [46,47,48,49].

The CpG4 site is located in the proximity to one of glucocorticoid response elements (GREs) at intron 7. It has been shown that activation of the glucocorticoid receptor after exposure to stress leads to demethylation of GREs and increased expression of the *FKBP5* gene [50,51]. Demethylation of GREs may further contribute to transcriptional effects of glucocorticoid receptors on the target genes [43].

Although cortisol levels were elevated in the group of patients, we found no significant correlations between the *FKBP5* methylation and cortisol levels after adjustment for potential confounding factors. This is consistent with the results of a recent study performed in healthy participants that controlled for the effects of various confounders [52]. However, it should also be noted that some studies have demonstrated a negative correlation between cortisol levels and the *FKBP5* methylation [44,53]. A lack of significant association between cortisol levels and the *FKBP5* methylation in the ANCOVA models suggests that our findings are not attributable to acute cortisol output.

It is important to note that we did not find any significant changes in the level of *FKBP5* methylation in patients with SCZ−AR after adjustment for potential confounding factors. Two scenarios should be taken into consideration when explaining this observation. Firstly, this group of patients is often characterized by a greater and longer exposure to various environmental factors that likely impact epigenetic processes, including various medications, comorbid physical health impairments and substance use. Indeed, previous studies also demonstrated that certain epigenetic alterations that appear in early psychosis cannot be observed in multiple−episode schizophrenia patients [54]. In turn, various confounding factors, including age, sex, cigarette smoking and BMI have previously been identified to impact the level of *FKBP5* methylation [43]. These factors were also related to *FKBP5* methylation in our sample. Another explanation might be associated with changes in biological responses to stress during subsequent exacerbation of psychosis. On the basis of a meta−analysis, Girshkin et al. (2014) found greater increases of morning cortisol levels in patients with established diagnosis of schizophrenia than those with FEP. In turn, our group demonstrated blunted release of neuroactive steroids during subsequent exacerbations of schizophrenia [55].

The present study demonstrated several clinical correlates of the *FKBP5* methylation. We found that lower *FKBP5* methylation, especially at CpG2 and CpG4 might be associated with better cognitive performance and general functioning in FEP and SCZ−AR patients. However, in healthy controls we found better performance of language skills and immediate memory in participants with higher CpG4 methylation. Increased expression of the *FKBP5* gene has been demonstrated in the hippocampus and prefrontal cortex of patients with schizophrenia [56,57]. Previous studies have also demonstrated that the FKBP51 and variation in its gene might be related to cognition. Szabó et al. [58] found better performance on the paired associates test, which is a sensitive measure of the hippocampus function, in patients with PTSD and higher expression of the *FKBP5* gene. The same group demonstrated a positive correlation between increases in the level of *FKBP5* gene expression and hippocampal volumes during cognitive-behavioural therapy in patients with PTSD [59]. Another study revealed that variation in the *FKBP5* gene (the rs1360870 polymorphism) might impact scores of the RBANS attention domain in patients with schizophrenia and matched controls as well as global cognition only in the group of patients with schizophrenia [28]. Altogether these findings suggest that hypomethylation of the *FKBP5* in early psychosis might be a protective mechanism against elevated cortisol levels. Indeed, enhanced *FKBP5* expression may lower glucocorticoid receptor sensitivity [25]. However, animal model studies have shown that the FKBP51 may worsen cognitive performance [24,60,61]. One of the potential mechanisms might be related to enhanced AMPA receptor recycling [60].

There are some important limitations to our study that need to be considered. Firstly, our sample was not large and thus the present findings need confirmation by larger studies. Secondly, we did not determine the level of the *FKBP5* expression. Therefore, conclusions regarding the functional impact of observed alterations cannot be established. Another important point is that we did not assess genetic variation in the *FKBP5* as allele-specific methylation changes have been reported for this gene [30]. Additionally, our analysis of cortisol levels was limited to a single morning measurement. Moreover, the majority of patients included in our study were not drug-naïve or drug-free, and recording the CPZeq might be insufficient to control for the medication effects. It is also important to note that assessment of ACEs with the use of retrospective measures might be characterized by a recall bias. Finally, a case-control study design does not allow us to establish causal associations.

## 5. Conclusions

In conclusion, results of this study indicate that decreased methylation of the *FKBP5* gene might be observed in patients at early stages of psychotic disorder. These alterations might be associated with better cognitive performance and general functioning in patients with psychosis but not healthy controls. A history of some ACEs, such as parental antipathy and sexual abuse might contribute to these alterations. Clinical and biological relevance as well as dynamics of the *FKBP5* methylation in this group of patients requires additional studies.

## Figures and Tables

**Table 1:** General characteristics of the sample.

	1. FEP		2. SCZ-AR		3. HCs		*p*	Post-Hoc or Pairwise Comparisons
	*n*	Mean ± SD or n (%)	*n*	Mean ± SD or n (%)	*n*	Mean ± SD or n (%)		
**Age, years**	40	28.1 ± 7.3	45	45.2 ± 12.6	56	32.5 ± 8.2	**<0.001**	1 < 2, 2 > 3
**Sex, males**	40	20 (50.0)	45	25 (55.6)	56	22 (39.3)	0.248	-
**Education, years**	40	13.6 ± 2.5	38	12.6 ± 3.0	54	15.8 ± 2.5	**<0.001**	1 < 3, 2 < 3
**Maternal education, higher**	40	8 (20.0)	45	8 (17.8)	53	18 (34.0)	0.130	-
**Paternal education, higher**	40	8 (20.0)	45	8 (17.8)	53	15 (28.3)	0.418	-
**BMI, kg/m^2^**	40	23.7 ± 3.8	40	26.5 ± 5.1	56	23.8 ± 3.5	**0.005**	1 < 2, 2 > 3
**Cigarette smoking**	40	15 (37.5)	40	22 (55.0)	56	5 (8.9)	**<0.001**	1 < 2, 1 > 3, 2 > 3
**Parental loss**	37	10 (27.0)	36	13 (36.1)	54	12 (22.2)	0.351	-
**Parental antipathy**	37	10 (27.0)	36	18 (50.0)	54	16 (57.1)	0.071	-
**Parental neglect**	37	6 (16.2)	36	13 (36.1)	54	14 (25.9)	0.153	-
**Physical abuse**	37	13 (35.1)	36	17 (47.2)	54	13 (24.1)	0.074	-
**Sexual abuse**	37	5 (13.5)	36	7 (19.4)	54	3 (5.6)	0.126	-
**RBANS–immediate memory**	40	42.7 ± 8.4	44	33.5 ± 11.3	52	51.9 ± 6.0	**<0.001**	1 < 3, 1 > 2, 2 < 3
**RBANS–visuospatial/constructional**	40	34.7 ± 5.4	44	30.0 ± 8.2	52	38.1 ± 2.2	**<0.001**	1 < 3, 1 > 2, 2 < 3
**RBANS–language**	40	28.2 ± 6.1	44	24.9 ± 6.6	52	33.7 ± 6.5	**<0.001**	1 < 3, 2 < 3
**RBANS–attention**	40	54.2 ± 12.2	44	35.6 ± 11.8	52	68.9 ± 8.9	**<0.001**	1 < 3, 1 > 2, 2 < 3
**RBANS–delayed memory**	40	46.9 ± 7.7	44	39.0 ± 11.3	52	56.0 ± 4.5	**<0.001**	1 < 3, 1 > 2, 2 < 3
**Age of psychosis onset, years**	40	26.6 ± 7.3	45	24.9 ± 8.6	-	-	0.109	-
**Illness duration, weeks**	40	43.8 ± 87.8	45	651.7 ± 526.9	-	-	**<0.001**	-
**Family history of psychosis**	40	5 (12.5)	45	12 (26.7)	56	0 (0)	**<0.001**	1 > 3, 2 > 3
**PANSS-P**	40	12.9 ± 5.2	40	15.2 ± 4.9	-	-	0.053	-
**PANSS-N**	40	18.1 ± 8.4	40	23.8 ± 9.5	-	-	**<0.001**	-
**MADRS**	40	8.3 ± 8.1	38	7.8 ± 8.3	-	-	0.743	-
**YMRS**	40	2.1 ± 5.1	38	2.1 ± 5.0	-	-	0.758	-
**GAF**	40	54.2 ± 17.1	39	35.3 ± 14.0	-	-	**<0.001**	-
**CPZeq, mg/day**	40	300.1 ± 169.7	37	467.7 ± 219.8	-	-	**<0.001**	-
**Cortisol, nmol/l**	40	338.6 73.3	45	448.9 151.9	55	272.8 ± 87.2	**<0.001**	1 < 2, 1 > 3, 2 > 3

Significant differences (*p* < 0.05) were marked with bold characters. n refers to the number of participants with available data.BMI, body mass index; CPZeq, chlorpromazine equivalent dosage; FEP, first-episode psychosis; GAF, the Global Assessment of Functioning; HCs, healthy controls; PANSS-N, the Positive and Negative Syndrome Scale (subscale of negative symptoms); MADRS, the Montgomery-Asberg Depression Rating Scale; PANSS-P, the Positive and Negative Syndrome Scale (subscale of positive symptoms); RBANS, the Repeatable Battery for the Assessment of Neuropsychological Status; SCZ-AR, acutely relapsed schizophrenia patients; YMRS, the Young Mania Rating Scale.

**Table 2:** The analysis of co-variance (ANCOVA) testing for the effects of diagnostic group (first-episode psychosis (FEP) vs. acutely relapsed schizophrenia (SCZ-AR) vs. controls) and childhood trauma on the *FKBP5* methylation.

*FKBP5*, %mC	Independent Variable	Parental Loss	Parental Antipathy	Parental Neglect	Physical Abuse	Sexual Abuse
**CpG1**	BMI	F = 3.380, *p* = 0.068	F = 3.336, *p* = 0.070	F = 3.561, *p* = 0.062	F = 3.263, *p* = 0.073	F = 3.030, *p* = 0.084
	Group	F = 1.749, *p* = 0.178	F = 1.765, *p* = 0.176	F = 2.702, *p* = 0.071	F = 2.239, *p* = 0.111	F = 0.422, *p* = 0.657
	ACEs	F = 1.076, *p* = 0.302	F = 0.007, *p* = 0.934	F = 0.536, *p* = 0.466	F = 0.489, *p* = 0.486	F = 0.854, *p* = 0.357
	Group × ACEs	F = 0.463, *p* = 0.631	F = 0.374, *p* = 0.689	F = 1.755, *p* = 0.177	F = 0.710, *p* = 0.494	F = 0.067, *p* = 0.935
**CpG2**	Age	F = 0.207, *p* = 0.650	F = 0.033, *p* = 0.856	F = 0.465, *p* = 0.497	F = 0.364, *p* = 0.548	F = 0.339, *p* = 0.562
	Sex	F = 2.521, *p* = 0.115	F = 2.616, *p* = 0.109	F = 3.025, *p* = 0.085	F = 2.780, *p* = 0.099	F = 3.024, *p* = 0.085
	Cortisol	F = 3.825, *p* = 0.053	F = 3.327, *p* = 0.071	F = 3.834, *p* = 0.052	F = 3.601, *p* = 0.061	F = 3.932, *p* = 0.058
	Group	F = 0.875, *p* = 0.420	F = 0.465, *p* = 0.629	F = 1.267, *p* = 0.286	F = 1.025, *p* = 0.362	F = 1.305, *p* = 0.276
	ACEs	F = 0.018, *p* = 0.894	F = 0.011, *p* = 0.916	F = 0.098, *p* = 0.755	F = 0.091, *p* = 0.764	F = 0.185, *p* = 0.668
	Group × ACEs	F = 1.243, *p* = 0.293	F = 1.882, *p* = 0.158	F = 0.153, *p* = 0.858	F = 1.518, *p* = 0.224	F = 0.867, *p* = 0.423
**CpG3**	Sex	**F = 19.934, *p* < 0.001**	**F = 21.481, *p* < 0.001**	**F = 20.618, *p* < 0.001**	**F = 22.292, *p* < 0.001**	**F = 21.116, *p* < 0.001**
	BMI	F = 1.988, *p* = 0.162	F = 2.744, *p* = 0.101	F = 1.601, *p* = 0.209	F = 1.831, *p* = 0.179	F = 2.056, *p* = 0.155
	Cigarette smoking	F = 0.446, *p* = 0.506	F = 0.528, *p* = 0.469	F = 0.509, *p* = 0.477	F = 0.531, *p* = 0.468	F = 0.235, *p* = 0.629
	Cortisol	F = 2.344, *p* = 0.129	F = 2.523, *p* = 0.115	F = 2.546, *p* = 0.114	F = 2.423, *p* = 0.123	F = 2.316, *p* = 0.131
	Group	F = 0.669, *p* = 0.514	F = 0.403, *p* = 0.670	F = 0.384, *p* = 0.682	F = 0.592, *p* = 0.555	F = 0.094, *p* = 0.911
	ACEs	F = 0.519, *p* = 0.473	F = 0.734, *p* = 0.394	F = 0.022, *p* = 0.883	F = 0.029, *p* = 0.864	F = 1.581, *p* = 0.211
	Group × ACEs	F = 0.253, *p* = 0.777	F = 0.047, *p* = 0.954	F = 0.669, *p* = 0.514	F = 0.935, *p* = 0.396	F = 1.104, *p* = 0.336
**CpG4**	Sex	**F = 22.416, *p* < 0.001**	**F = 25.458, *p* < 0.001**	**F = 21.119, *p* < 0.001**	**F = 25.114, *p* < 0.001**	**F = 26.495, *p* < 0.001**
	BMI	**F = 4.020, *p* = 0.047**	**F = 6.733,** *p* **= 0.011**	F = 3.813, *p* = 0.053	**F = 4.038, *p* = 0.047**	**F = 4.556, *p* = 0.035**
	CPZeq	F = 0.004, *p* = 0.952	F = 0.372, *p* = 0.543	F = 0.078, *p* = 0.780	F = 0.401, *p* = 0.528	F = 0.009, *p* = 0.923
	Group	**F = 6.247, *p* = 0.003**	**F = 8.302, *p* < 0.001**	**F = 6.147, *p* = 0.003**	**F = 6.929, *p* = 0.001**	**F = 5.994, *p* = 0.003**
	ACEs	F = 1.040, *p* = 0.310	**F = 5.956, *p* = 0.016**	F = 0.134, *p* = 0.715	F = 0.023, *p* = 0.880	**F = 5.470, *p* = 0.021**
	Group × ACEs	F = 1.430, *p* = 0.244	F = 0.269, *p* = 0.765	F = 0.195, *p* = 0.823	F = 2.164, *p* = 0.120	F = 0.068, *p* = 0.934

Significant effects (*p* < 0.05) were marked with bold characters. ACEs, adverse childhood experiences.

**Table 3:** Correlations between clinical variables and the *FKBP5* methylation.

	FEP				SCZ-AR				HCs			
	CpG1	CpG2	CpG3	CpG4	CpG1	CpG2	CpG3	CpG4	CpG1	CpG2	CpG3	CpG4
**RBANS–immediate memory**	*r* = −0.198 *p* = 0.222	***r* = −0.354** ***p* = 0.025**	*r* = −0.039 *p* = 0.810	***r* = −0.310** ***p* = 0.049**	*r* = −0.224 *p* = 0.143	***r* = −0.398** ***p* = 0.008**	*r* = −0.029 *p* = 0.850	***r* = −0.480** ***p* = 0.001**	*r* = 0.150 *p* = 0.287	*r* = 0.081 *p* = 0.570	*r* = 0.037 *p* = 0.795	***r* = 0.289** ***p* = 0.038**
**RBANS–visuospatial/constructional**	*r* = 0.120 *p* = 0.461	***r* = −0.442** ***p* = 0.004**	*r* = −0.007 *p* = 0.964	*r* = −0.128 *p* = 0.430	*r* = 0.218 *p* = 0.156	*r* = 0.012 *p* = 0.940	*r* = −0.061 *p* = 0.695	*r* = 0.122 *p* = 0.431	*r* = 0.023 *p* = 0.872	*r* = 0.088 *p* = 0.536	*r* = −0.250 *p* = 0.073	*r* = 0.102 *p* = 0.473
**RBANS–language**	*r* = 0.052 *p* = 0.748	*r* = −0.259 *p* = 0.106	*r* = 0.264 *p* = 0.100	*r* = 0.074 *p* = 0.649	*r* = −0.231 *p* = 0.132	*r* = −0.013 *p* = 0.932	*r* = −0.133 *p* = 0.389	*r* = −0.091 *p* = 0.557	*r* = −0.001 *p* = 0.993	*r* = −0.101 *p* = 0.477	*r* = 0.227 *p* = 0.106	***r* = 0.277** ***p* = 0.047**
**RBANS–attention**	*r* = 0.172 *p* = 0.289	***r* = −0.353** ***p* = 0.026**	*r* = 0.050 *p* = 0.758	***r* = −0.348** ***p* = 0.028**	*r* = −0.173 *p* = 0.261	***r* = −0.464** ***p* = 0.001**	*r* = 0.074 *p* = 0.634	***r* = −0.452** ***p* = 0.002**	*r* = −0.027 *p* = 0.847	*r* = −0.146 *p* = 0.303	*r* = 0.243 *p* = 0.083	*r* = 0.170 *p* = 0.230
**RBANS–delayed memory**	*r* = 0.050 *p* = 0.760	***r* = −0.340** ***p* = 0.032**	*r* = −0.165 *p* = 0.308	***r* = −0.340** ***p* = 0.032**	*r* = 0.006 *p* = 0.970	***r* = −0.333** ***p* = 0.027**	*r* = −0.061 *p* = 0.693	***r* = −0.460** ***p* = 0.002**	*r* = 0.149 *p* = 0.291	*r* = 0.140 *p* = 0.322	*r* = −0.017 *p* = 0.907	*r* = 0.250 *p* = 0.074
**PANSS−P**	*r* = 0.043 *p* = 0.791	*r* = 0.147 *p* = 0.366	*r* = −0.070 *p* = 0.667	*r* = 0.051 *p* = 0.756	*r* = 0.103 *p* = 0.526	*r* = 0.262 *p* = 0.102	*r* = 0.039 *p* = 0.813	*r* = 0.014 *p* = 0.930	−	−	−	−
**PANSS−N**	*r* = 0.079 *p* = 0.627	*r* = −0.104 *p* = 0.525	*r* = −0.160 *p* = 0.323	*r* = −0.129 *p* = 0.427	*r* = 0.014 *p* = 0.993	*r* = 0.042 *p* = 0.799	*r* = 0.087 *p* = 0.595	*r* = −0.087 *p* = 0.594	−	−	−	−
**MADRS**	*r* = 0.175 *p* = 0.280	*r* = 0.057 *p* = 0.725	*r* = −0.135 *p* = 0.406	*r* = 0.003 *p* = 0.986	*r* = 0.253 *p* = 0.126	*r* = 0.256 *p* = 0.121	*r* = 0.087 *p* = 0.605	*r* = 0.007 *p* = 0.966	−	−	−	−
**YMRS**	*r* = −0.019 *p* = 0.908	*r* = 0.166 *p* = 0.306	*r* = 0.039 *p* = 0.812	*r* = 0.079 *p* = 0.626	*r* = 0.265 *p* = 0.107	*r* = 0.033 *p* = 0.845	*r* = 0.052 *p* = 0.755	*r* = 0.192 *p* = 0.248	−	−	−	−
**GAF**	*r* = 0.165 *p* = 0.310	***r* = −0.442** ***p* = 0.004**	*r* = 0.053 *p* = 0.746	***r* = −0.346** ***p* = 0.029**	*r* = 0.041 *p* = 0.805	***r* = −0.471** ***p* = 0.003**	*r* = −0.175 *p* = 0.287	***r* = −0.314** ***p* = 0.048**	−	−	−	−

Significant correlations (*p* < 0.05) were marked with bold characters. FEP, first-episode psychosis; GAF, the Global Assessment of Functioning; HCs, healthy controls; PANSS-N, the Positive and Negative Syndrome Scale (subscale of negative symptoms); MADRS, the Montgomery-Asberg Depression Rating Scale; PANSS-P, the Positive and Negative Syndrome Scale (subscale of positive symptoms); RBANS, the Repeatable Battery for the Assessment of Neuropsychological Status; SCZ-AR, acutely relapsed schizophrenia patients; YMRS, the Young Mania Rating Scale.

## Appendix A

**Table A1:** Correlations between potential confounding factors and the level of methylation at tested FKBP5 CpG sites in the whole sample.

	CpG1	CpG2	CpG3	CpG4
Age	*r* = 0.072, *p* = 0.396	***r* = 0.167, *p* = 0.048**	*r* = −0.092, *p* = 0.277	*r* = 0.156, *p* = 0.064
Sex	Males vs. females: 96.6 ± 3.6 vs. 96.4 ± 3.4, *p* = 0.617	Males vs. females: 99.3 ± 1.2 vs. 99.7 ± 0.9, ***p* = 0.048**	Males vs. females: 63.9 ± 4.2 vs. 67.1 ± 4.5, ***p* < 0.001**	Males vs. females: 64.6 ± 6.8 vs. 69.1 ± 8.3, ***p* = 0.002**
Cigarette smoking	Non−smokers vs. smokers: 96.3 ± 3.6 vs. 96.7 ± 3.0, *p* = 0.684	Non−smokers vs. smokers: 99.6 ± 1.0 vs. 99.3 ± 1.2 *p* = 0.107	Non−smokers vs. smokers: 66.1 ± 4.4 vs. 64.2 ± 5.0, ***p* = 0.035**	Non−smokers vs. smokers: 67.1 ± 7.9 vs. 65.9 ± 8.0, *p* = 0.330
BMI	*r* = 0.171, ***p* = 0.047**	*r* = 0.070, *p* = 0.421	***r* = 0.212, *p* = 0.013**	***r* = 0.212, *p* = 0.013**
CPZeq	*r* = 0.139, *p* = 0.227	*r* = 0.166, *p* = 0.149	*r* = 0.068, *p* = 0.232	***r* = 0.262, *p* = 0.022**
Cortisol	*r* = 0.167, *p* = 0.070	***r* = 0.267, *p* = 0.003**	***r* = −0.198, *p* = 0.031**	*r* = 0.086, *p* = 0.355

Significant associations (*p* < 0.05) were marked with bold characters.
